# A prognostic model for platinum‐doublet as second‐line chemotherapy in advanced non‐small‐cell lung cancer patients

**DOI:** 10.1002/cam4.689

**Published:** 2016-03-19

**Authors:** Hongnan Mo, Xuezhi Hao, Yutao Liu, Lin Wang, Xingsheng Hu, Jianping Xu, Sheng Yang, Puyuan Xing, Youwu Shi, Bo Jia, Yan Wang, Junling Li, Hongyu Wang, Ziping Wang, Yan Sun, Yuankai Shi

**Affiliations:** ^1^ Department of Medical OncologyCancer Hospital, Chinese Academy of Medical Sciences (CAMS) & Peking Union Medical College (PUMC); Beijing Key Laboratory of Clinical Study on Anticancer Molecular Targeted DrugsBeijingChina

**Keywords:** Antineoplastic combined chemotherapy protocols, Carcinoma, nomograms, Non‐small‐cell lung, prognosis, treatment outcome

## Abstract

Poor prognosis of advanced non‐small‐cell lung cancer (NSCLC) patients and the promising therapeutic effect of platinum urge the oncologists to evaluate the role of platinum doublet as second‐line chemotherapy and establish the definition of platinum sensitivity in NSCLC. We retrospectively analyzed 364 advanced NSCLC patients who received platinum‐doublet regimens as second‐line chemotherapy after platinum‐based first‐line treatment. Patients were divided into four groups by their time‐to‐progression (TTP) after first‐line chemotherapy: 0–3, 4–6, 7–12, and >12‐month group, respectively. Treatment efficacy of patients' overall survival (OS), progression‐free survival (PFS), and response rate (RR), as well as treatment‐related toxicity, were compared among the four groups. A prognosis score system and a nomogram were established by Cox proportional hazard model, and validated by concordance index (c‐index). Median OS was 14.0, 16.0, 20.0, 25.0 months for patients in the 0–3, 4–6, 7–12, >12‐month group, respectively. Age ≤60 years (*P = *0.002), female (*P = *0.019), and TTP>12 months (*P = *0.003) were independent prognostic factors. Prognostic score was calculated by adding 1 point each for any of the above three indicators, with a c‐index of 0.590 (95% confidential interval [CI], 0.552–0.627). Median OS were equal to 25.0, 16.0, and 11.0 months for best (2–3 points), intermediate (1 point) and worst (0 point) category, respectively (*P < *0.0001). A nomogram that integrated patient's age, gender, and TTP for OS has a c‐index of 0.623 (95% CI, 0.603–0.643). Female, younger than 60 years, and TTP greater than 12 months may indicate prolonged survival after platinum‐doublet second‐line chemotherapy in advanced NSCLCpatients.

## Introduction

Lung cancer is the leading causes of cancer death worldwide. Non‐small‐cell lung cancer (NSCLC) accounts for more than 80% of all lung cancer cases. First‐line treatment for stage IIIB and IV NSCLC patients usually consists of a platinum‐doublet chemotherapy 1. Unfortunately, all of the advanced NSCLC patients will inevitably experience disease progression after first‐line therapy. At that time, a substantial part of these patients may still have a good performance status (PS), and be eligible for further anticancer treatment. Certain prognostic factors for second‐line treatment of advanced NSCLC include disease stage, PS, female gender, and previous response to first‐line treatment 2.

Single‐agent chemotherapy, including docetaxel 3, pemetrexed 4, and tyrosine kinase inhibitors of the epidermal growth factor receptor (EGFR‐TKI, including gefitinib 5, erlotinib 6, and icotinib 7), is approved for second‐line treatment in advanced NSCLC patients with a PS of 0–2. Meanwhile, prognosis of these patients remains poor, with a response rate (RR) of less than 10% and a median survival of only 6–8 months 3, 4, 6. This challenges the oncologists to explore for novel treatment strategies for each specific patient. Platinum combination chemotherapy seem to be promising candidates, but their role in second‐line treatment of advanced NSCLC patients is still a matter of debate.

Di Maio et al. analyzed six randomized trials comparing single agent with doublet chemotherapy as second‐line treatment in advanced NSCLC 8. They found that doublet chemotherapy resulted in more toxicity compared to single agent and did not increase the overall survival (OS) of advanced NSCLC patients. However, this meta‐analysis only included four clinical trials that enrolled patients pretreated with platinum‐based chemotherapy, and only one of them compared platinum doublet to a single agent as second‐line chemotherapy.

Platinum sensitivity has been well established in many tumors, including small‐cell lung cancer (SCLC) and ovarian cancer. Platinum rechallenge means platinum‐based second‐line chemotherapy in patients pretreated with platinum‐based chemotherapy. In SCLC, the platinum‐sensitive group includes patients who have a time‐to‐progression (TTP) of at least 3 months, and platinum rechallenge second‐line chemotherapy results in better progression‐free survival (PFS) and OS 9, 10. In ovarian cancer, disease that recurs more than 6 months after prior therapy is categorized as “platinum sensitive,” and platinum‐based therapy is the principal regimen for it in second‐line treatment 11.

The role of platinum rechallenge in advanced NSCLC patients has not been well evaluated. A pooled analysis of 11 studies showed that in patients pretreated with platinum‐based chemotherapy, the RR of platinum doublet as second‐line treatment was 27.5%, with a median PFS of 3.9 months, and a median OS of 8.7 months 12. Results of the GOIRC 02‐2006 study concluded that patients pretreated with platinum doublet did not benefit from the addition of carboplatin to pemetrexed in second‐line chemotherapy 13. Instead of “TTP after first‐line treatment,” platinum sensitivity was interpreted by “response to first‐line platinum‐doublet” and “treatment‐free interval” in this trial, and neither of them predicted the efficacy of second‐line treatment. The NVALT 7 trial compared pemetrexed plus carboplatin with pemetrexed as second‐line chemotherapy in patients relapsing more than 3 months after platinum‐based chemotherapy 14. Interestingly, there was significantly improved OS for patients who had previous treatment more than 6 months before. Arrieta et al. analyzed platinum rechallenge in 23 stage IV NSCLC patients who experiencing disease progression 6 months after platinum‐based first‐line chemotherapy, resulting a RR of 30.4%, a median PFS of 5.9 months, and a median OS of 12.5 months 15. Lee et al. evaluated the efficacy of pemetrexed plus oxaliplatin in platinum‐resistant advanced NSLCL patients, and not surprisingly, no response was observed 16.

In NSCLC, the definition of “platinum sensitivity” has not been clearly established yet, and there are few studies that focus on TTP after the first‐line treatment when they evaluate the efficacy of platinum rechallenge as second‐line treatment. This is the first study aiming to define a prognostic score for platinum sensitivity in advanced NSCLC patients, and to assess the clinical efficacy of platinum doublet as second‐line chemotherapy.

## Methods

### Identification of eligible patients

We retrospectively collected data from 364 consecutive, unselected advanced NSCLC patients, who were admitted to Cancer Institute/ Hospital, Chinese Academy of Medical Sciences & Peking Union Medical College between February 1999 and April 2014.

Patients eligible for this trial were required to meet the following criteria: (1) histologically or cytologically confirmed NSCLC with stage IIIB or IV disease; (2) had platinum‐doublet chemotherapy as first‐line treatment and disease progression; (3) received platinum‐doublet chemotherapy as second‐line treatment.

Patients were further divided into four groups by their TTP after first‐line chemotherapy: 0–3 months group, 4–6 months group, 7–12 months group, and >12 months group. Cutoffs were chosen according to previous literature and at equal distance along the range of values 14, 15. Data collected included disease stage at diagnosis, first‐line regimen received, response to first‐line treatment, TTP after first‐line treatment, demographic characteristics before second‐line treatment, Eastern Cooperative Oncology Group (ECOG) PS before second‐line treatment, type of second‐line regimen, chemotherapy‐related toxicity during second‐line treatment, response to second‐line treatment, PFS and OS after second‐line treatment.

Approval for the retrospective review of these patients' records was obtained from the Institutional Review Board of the hospital.

### Treatment and response assessment

All patients were treated by platinum‐based second‐line chemotherapy until disease progression, or for a maximum of six cycles. Chemotherapy‐related toxicities were recorded according to the National Cancer Institute Common Terminology Criteria for Adverse Events (CTCAE 4.0). Radiological response was assessed by treating physicians every two chemotherapy episodes according to the Response Evaluation Criteria in Solid Tumors (1.1). Objective response is defined as complete response (CR) and partial response (PR). TTP after first‐line chemotherapy was calculated from date of initiation of first‐line chemotherapy until disease progression. PFS after second‐line therapy was defined as the time between the date of initiation of second‐line chemotherapy and the date of disease progression or patient death. OS was defined as the time from the beginning of the second‐line chemotherapy until patient death resulting from any cause. Living patients were censored at the date of last follow‐up.

### Statistical analysis

Categorical variables were compared using the chi‐square test or paired chi‐square test, when appropriate. PFS and OS probabilities were computed according to the method of Kaplan–Meier and Cox regression. Hazard ratios (HRs) with 95% CI were calculated with Cox proportional hazards models. Survival rates were calculated by the Kaplan–Meier method. A P value less than 0.05 was considered to be statistically significant. The statistical analyses were performed using SPSS version 18.0 (SPSS Inc., Chicago, IL).

We established a novel prognostic score system, TAF score, for platinum sensitivity assessment in advanced NSCLC. Significant factors (*P* < 0.1) from univariate selection were kept in the multivariate analysis. Coefficients were assigned proportionally to the HRs of the significant factors in the final prognostic score system 2. A prognostic nomogram was formulated by using the rms package in R (http://www.r-project.org/). The predictive validity of the TAF score system and the nomogram were accessed by the Harrell's c statistic (concordance index, C‐index) and the bootstrap analysis (1000 samples), using the package of the rcorrp.cens package in Hmisc in R 17, 18, 19.

## Results

### Patient characteristics

Data were collected from 364 advanced NSCLC patients who had failed first‐line platinum‐doublet chemotherapy, and received platinum‐doublet chemotherapy as second‐line treatment from February 1999 to April 2014. The patients were categorized according to their TTP after the first‐line treatment. Baseline characteristics are listed in Table 1. One hundred seventy‐nine (49.2%) patients had disease progression within 3 months after the first‐line chemotherapy, 94 (25.8%) patients in the 4‐ to 6‐month group, 60 (16.5%) patients in the 7‐ to 12‐month group, and 31 (8.5%) patients in the >12‐month group.

**Table 1:** Patient and tumor characteristics by time‐to‐progression after first‐line chemotherapy

	0–3 months (*N *=* *179)	4–6 months (*N *=* *94)	7–12 months (*N *=* *60)	>12 months (*N *=* *31)	All (*N *=* *364)
Age (years)					
Median, range	54,21–73	55,32–73	56,29–73	57,37–76	55,21–76
<60 years	131 (73.2%)	69 (73.4%)	39 (65.0%)	18 (58.1%)	257 (70.6%)
≥60 years	48 (26.8%)	25 (26.6%)	21 (35.0%)	13 (41.9%)	207 (29.4%)
Gender					
Male	135 (75.4%)	71 (75.5%)	43 (71.7%)	24 (77.4%)	273 (75.0%)
Female	44 (24.6%)	23 (24.5%)	17 (28.3%)	7 (22.6%)	91 (25.0%)
ECOG PS					
0	123 (68.7%)	56 (59.6%)	37 (61.7%)	22 (71.0%)	238 (65.4%)
1	56 (31.3%)	38 (40.4%)	23 (38.3%)	9 (29.0%)	126 (34.6%)
Stage					
IIIB	29 (16.2%)	25 (26.6%)	19 (31.7%)	11 (35.5%)	84 (23.1%)
IV	150 (83.8%)	69 (73.4%)	41 (68.3%)	20 (64.5%)	280 (76.9%)
First‐line response					
CR/PR	17 (9.5%)	49 (52.1%)	32 (53.3%)	18 (58.1%)	116 (31.9%)
SD	90 (50.3%)	43 (45.7%)	27 (45.0%)	12 (38.7%)	172 (47.3%)
PD	72 (40.2%)	2 (2.1%)	1 (1.7%)	1 (3.2%)	76 (20.9%)
Histology					
Adenocarcinoma	133 (74.3%)	51 (54.3%)	32 (53.3%)	14 (45.2%)	230 (63.2%)
Squamous	30 (16.8%)	31 (33.0%)	17 (28.3%)	15 (48.4%)	93 (25.5%)
Large cell	1 (0.6%)	3 (3.2%)	2 (3.3%)	0 (0%)	6 (1.6%)
NOS	15 (8.4%)	9 (9.6%)	9 (15.0%)	2 (6.5%)	35 (9.6%)
First‐line regimen					
Pt + Paclitaxel	88 (49.2%)	38 (40.4%)	36 (60.0%)	18 (58.1%)	180 (49.5%)
Pt + Gemcitabine	48 (26.8%)	23 (24.5%)	14 (23.3%)	7 (22.6%)	92 (25.3%)
Pt + Pemetrexed	21 (11.7%)	6 (6.4%)	2 (3.3%)	2 (6.5%)	31 (8.5%)
Pt + Vinorelbine	16 (8.9%)	20 (21.3)	6 (10.0%)	3 (9.7%)	45 (12.4%)
Pt + Etoposide	4 (2.2%)	4 (4.3%)	1 (1.7%)	1 (3.2%)	10 (2.7%)
Pt + Irinotecan	2 (1.1%)	3 (3.2%)	1 (1.7%)	0 (0%)	6 (1.6%)
Second‐line regimen					
Pt + Paclitaxel	68 (38.0%)	44 (46.8%)	21 (35%)	12 (38.7%)	145 (39.8%)
Pt + Gemcitabine	37 (20.7%)	21 (22.3%)	14 (23.3)	9 (29.0%)	81 (22.3%)
Pt + Pemetrexed	62 (34.6%)	14 (14.9%)	15 (25.0%)	8 (25.8%)	99 (27.2%)
Pt + Vinorelbine	9 (5.0%)	13 (13.8)	8 (13.3%)	2 (6.5%)	32 (8.8%)
Pt + Etoposide	2 (1.1%)	0 (0%)	2 (3.3%)	0 (0%)	4 (1.1%)
Pt + Irinotecan	1 (0.6%)	2 (2.1%)	0 (0%)	0 (0%)	3 (0.8%)

ECOG, Eastern Cooperative Oncology Group; PS, performance status; CR, complete response; PR, partial response; SD, stable disease; PD, progressive disease; NOS, not otherwise specified; Pt, platinum.

Median age was 55 years (range, 21–76 years) when patient began the second‐line treatment. All patients had ECOG PS of 0 or 1. Two hundred seventy‐three (75.0%) patients were men, 238 (65.4%) had ECOG PS of 0, 280 (76.9%) had an initial diagnosis of stage IV disease, and 230 (63.2%) were diagnosed with adenocarcinoma. Tumors were adenocarcinomas in 59.0% of males compared to 75.8% of females, while squamous carcinomas were more common among men than women (29.7% and 13.2%, respectively). The four treatment groups were well balanced for patient age, gender, and PS (*P* = 0.242, 0.923, and 0.381, respectively), with the exception of clinical stage and histology: patients whose disease progressed more than 12 months after first‐line chemotherapy had less stage IV disease (*P* = 0.014) and more squamous cell carcinoma (*P* = 0.000) than the other three groups.

As first‐line chemotherapy, 180 (49.5%) patients had received platinum plus paclitaxel, 92 (25.3%) patients had received platinum plus gemcitabine, 31 (8.5%) patients had received platinum plus pemetrexed, 45 (12.4%) patients had received platinum plus vinorelbine, 10 (2.7%) patients had received platinum plus etoposide, and 6 (1.6%) patients had received platinum plus irinotecan. The median (range) number of first‐line chemotherapy cycles was 2 (1–6), 4 (1–6), 4 (2–8), and 4 (2–8) in the 0–3, 4–6, 7–12, >12‐month group, respectively. Overall, 116 (31.9%) of patients had achieved CR/PR to first‐line treatment. This proportion varied significantly in the different groups, ranging from 9.5%, 52.1%, 53.3% to 58.1% (*P* < 0.0001).

One hundred forty‐five (39.8%) of all patients in second‐line treatment had received platinum plus paclitaxel, 81 (22.3%) patients had received platinum plus gemcitabine, 99 (27.2%) patients had received platinum plus pemetrexed, 32 (8.8%) patients had received platinum plus vinorelbine, four (1.1%) patients had received platinum plus etoposide, and three (0.8%) patients had received platinum plus irinotecan. The median number of second‐line chemotherapy cycles was 2 (range, 1–10) in all patients, two cycles (range, 1–10) in the 0‐ to 3‐month group, three cycles (range, 1–6) in the 4‐ to 6‐month group, three cycles (range, 1–6) in the 7‐ to 12‐month group and four cycles (range, 1–6) in the >12‐month group, respectively.

### Chemotherapy‐related toxicity in second‐line treatment

The frequency of treatment‐related toxicity exceeding CTCAE grade 2 was less than 5% for all categories, with the exception of hematologic toxicity (Table 2). No episode of death or treatment delay was recorded during the study. The most frequent grade 3/4 hematological toxicity was neutropenia, which occurred in 29 (16.2%) patients of the 0‐ to 3‐month group, 25 (26.6%) patients of the 4‐ to 6‐month group, 16 (26.6%) patients of the 7‐ to 12‐month group, and three (9.7%) patients of the >12‐month group (*P* = 0.048). Nausea/vomiting were the most frequent observed grade 3/4 nonhematological toxicity, which were observed in eight (4.5%) patients of the 0‐ to 3‐month group, three (3.2%) patients of the 4‐ to 6‐month group, 0 (0%) patients of the 7‐ to 12 month group, and one (3.2%) patient of the >12‐month group (*P* = 0.420). Nineteen (5.2%) patients experienced grade 1/2 neuropathy, which was tolerable and did not result in treatment delay.

**Table 2:** Grade 3 and 4 toxicities of second‐line platinum‐based chemotherapy

	0–3 months (*N *=* *179)	4–6 months (*N *=* *94)	7–12 months (*N *=* *60)	>12 months (*N *=* *31)	All (*N *=* *364)
Hematological toxicity					
Anemia	6 (3.3%)	1 (1.1%)	2 (3.3%)	0 (0%)	9 (2.5%)
Neutropenia	29 (16.2%)	25 (26.6%)	16 (26.6%)	3 (9.7%)	73 (20.1%)
Thrombocytopenia	7 (3.9%)	8 (8.5%)	6 (10.0%)	2 (6.5%)	23 (6.3%)
Nonhematological toxicity					
Fever	1 (0.5%)	1 (1.1%)	0 (0%)	0 (0%)	2 (0.5%)
Nausea/vomit	8 (4.5%)	3 (3.2%)	0 (0%)	1 (3.2%)	12 (3.3%)
Alopecia	1 (0.5%)	1 (1.1%)	0 (0%)	0 (0%)	2 (0.5%)
Neuropathy	0 (0%)	0 (0%)	0 (0%)	0 (0%)	0 (0%)

### Response to second‐line treatment

RR of the second‐line treatment was similar among the four groups (10.6% vs. 8.5% vs. 18.3% vs. 12.9%, respectively; *P* = 0.288). Three complete remissions were observed in the 7‐ to 12‐month group, while 19, eight, and four patients had a confirmed partial remission as best response in the 0–3, 4–6, and >12‐month group, respectively (Table 3). Patients progressing less than 3 months after the first‐line platinum‐based chemotherapy tended to have more progressive disease (45 patients [25.1%]; *P* = 0.266) when compared with the other three groups (24 patients [25.5%] vs. 10 patients [16.7%] vs. 4 patients [12.9%], respectively). The median duration of response was 3.0 months: 2.0 months (95% CI, 1.1–2.9) for the 0‐ to 3‐month group, 3.0 months (95% CI, 2.0–4.0) for the 4‐ to 6‐month group, 4.0 months (95% CI, 2.8–5.2) for the 7‐ to 12‐month group, and 5.0 months (95% CI, 2.7–7.2) for the >12‐month group (*P* = 0.021), respectively. Patients who had CR or PR in the first‐line platinum doublet was more likely to respond to the second‐line platinum‐based chemotherapy (17/116 [14.7%] vs. 25/248 [10.1%], *P* < 0.0001).

**Table 3:** Disease response and patient survival after second‐line treatment

	0–3 months (*N *=* *179)	4–6 months (*N *=* *94)	7–12 months (*N *=* *60)	>12 months (*N *=* *31)	All (*N *=* *364)
Best response					
CR/PR	19 (10.6%)	8 (8.5%)	11 (18.3%)	4 (12.9%)	42 (11.5%)
SD	87 (48.6%)	50 (53.2%)	32 (53.3%)	21 (67.7%)	190 (52.2%)
PD	45 (25.1%)	24 (25.5%)	10 (16.7%)	4 (12.9%)	83 (22.8%)
NA	28 (15.6%)	12 (12.8%)	7 (11.7%)	2 (6.5%)	49 (13.5%)
PFS					
Median (months)	2.0	3.0	4.0	5.0	3.0
95% CI	1.1–2.9	2.0–4.0	2.8–5.2	2.7–7.2	2.3–3.7
Stratified HR					0.851
95% CI					0.746–0.970
OS					
Median (months)	14.0	16.0	20.0	25.0	16.0
95% CI	11.5–16.5	13.8–18.1	15.4–24.6	18.1–31.9	13.5–18.5
Stratified HR					0.809
95% CI					0.703–0.931
1‐Year survival					
Estimated rate (%)	55.4	59.8	64.4	80.9	60.4
95% CI	47.2–63.6	48.8–70.8	51.9–76.9	65.8–96.0	54.9–65.9
2‐Year survival					
Estimated rate (%)	27.8	39.6	35.4	51.6	34.2
95% CI	19.8–35.8	28.0–51.2	21.7–49.1	28.7–74.5	28.3–40.1
3‐Year survival					
Estimated rate (%)	20.2	28.5	19.7	36.8	23.7
95% CI	12.4–28.0	16.7–40.3	6.0–33.4	12.9–60.7	17.8–29.6
Follow‐up					
Median (months)					11.0
95% CI					1.0–63.6

CR, complete response; PR, partial response; SD, stable disease; PD, progressive disease; NA, not assessable; PFS, progress‐free survival; OS, overall survival; CI, confidential interval; HR, hazard ratio.

**Figure 1:**
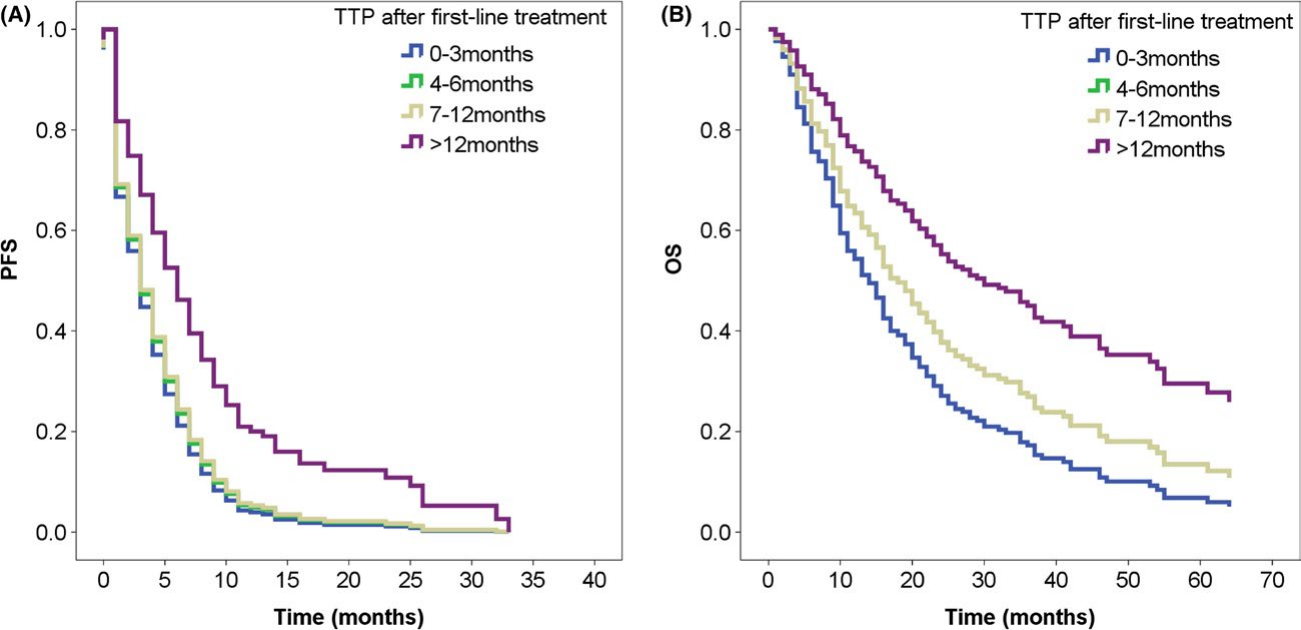
Progression‐free survival (PFS) and overall survival (OS) survival curve by time‐to‐progression (TTP) after second‐line treatment according to the method of Cox regression. (A) Patients in the >12‐month group had significant better PFS compared with the remaining three groups (*P* = 0.016). (B) Patients in the >12‐month group had the best OS after second‐line platinum‐based treatment (*P* = 0.003), while long‐term survival of patients in the 4‐ to 6‐month group were almost the same as that of patients in the 7‐ to 12‐month group.

The median follow‐up time after second‐line treatment was 11.0 months. Median PFS after initiation of second‐line chemotherapy was 2.0 months (95% CI, 1.1–2.9 months) for patients in the 0‐ to 3‐month group, 3.0 months (95% CI, 2.0–4.0 months) for those in the 4‐ to 6‐month group, 4.0 months (95% CI, 2.8–5.2 months) for those in the 7‐ to 12‐month group, and 5.0 months (95% CI, 2.7–7.2 months) for those in the >12‐month group (*P* = 0.021). The HR for disease progression was 0.851 (95% CI, 0.756–0.970) in favor of patients in the >12‐month group (*P* = 0.016, Fig. 1A). Of the 364 patients recruited, eight patients were lost to follow‐up, and 219 patients died, all of whom were cancer‐related deaths. Median OS from the beginning of second‐line chemotherapy was 14.0 months (95% CI, 11.5–16.5 months) versus 16.0 months (95% CI, 13.8–18.1 months) versus 20.0 months (95% CI, 15.4–24.6 months) versus 25.0 months (95% CI, 18.1–31.9 months) for patients in the 0–3, 4–6, 7–12, >12‐month group, respectively. Patients with TTP > 12 months had significant longer survival than the rest of the group (HR, 0.809; 95% CI, 0.703–0.931; *P* = 0.003; Fig. 1B). As compared with patients with a TTP of 0–3 months after first‐line treatment, HR was 0.746 (95% CI, 0.540–1.031; *P* = 0.076) for patients in the 4‐ to 6‐month group, 0.746 (95% CI, 0.518–1.075; *P* = 0.115) for patients in the 7‐ to 12‐month group, and 0.454 (95% CI, 0.256–0.807; *P* = 0.007) for the >12‐month group. The estimated 1‐year survival rate for all patients was 60.4% (95% CI, 54.9–65.9), with no discernible difference among the four groups (*P* = 0.165). The estimated 3‐year survival rate was 20.2%, 28.5%, 19.7%, and 36.8% for the 0–3, 4–6, 7–12, >12‐month group, respectively (*P* = 0.161).

### Prognostic TAF Score for OS

The univariate analysis revealed that age (*P* = 0.000), gender (*P* = 0.004), and TTP after first‐line treatment (*P* = 0.042) were significant prognostic factors for patient survival, whereas ECOG PS (*P* = 0.415) and objective response of first‐line chemotherapy did not (*P* = 0.440). Disease stage (*P* = 0.145) and tumor histology (*P* = 0.174) had only borderline significance (Table 4). Age was coded as >60 or ≤60 years because this dichotomy was most commonly used in precious analysis. As we mentioned above, patients in the >12‐month group had significant better OS compared with the rest three groups. TTP after first‐line treatment was categorized into 0–12 and >12 months.

**Table 4:** Univariate and multivariate analysis

	Univariate analysis	Multivariate analysis	
	*P* value	Hazard ratio (95% CI)	*P* value
Age <60 years	0.000	0.633 (0.477–0.841)	0.002
Female	0.004	0.681 (0.493–0.939)	0.019
TTP >12 months	0.042	0.806 (0.700–0.928)	0.003
Disease stage	0.145		0.144
Tumor histology	0.174		0.173
ECOG PS	0.415		
objective response of 1st chemotherapy	0.440		

ECOG, Eastern Cooperative Oncology Group; PS, performance status; TTP, time‐to‐progression after first‐line chemotherapy.

Patient's age, gender, TTP after first‐line chemotherapy, disease stage and tumor histology were included in the multivariate analysis. Long‐term survival was better for patients younger than 60 years (HR 0.633, 95% CI, 0.477–0.841, *P* = 0.002), female (HR 0.681, 95% CI, 0.493–0.939, *P* = 0.019), and TTP>12 months (HR 0.806, 95% CI, 0.700–0.928, *P* = 0.003). Disease stage and tumor histology were not independent factors of patient's survival.

We calculated TAF Score by adding 1 point each for any of the following: TTP of more than 12 months after first‐line treatment, age ≤60 years, and female. Groups were defined by comparing the HRs in patients with each possible number of points (0, 1, 2, or 3). The categories of 2 and 3 were combined because of similar HRs. Three distinct prognostic groups were formed based on Kaplan–Meier curves: worse (0 point), intermediate (1 point), and best (2 or 3 points). This three‐category score exhibited a C‐index estimate equal to 0.590 (95%CI, 0.552–0.627).

**Figure 2:**
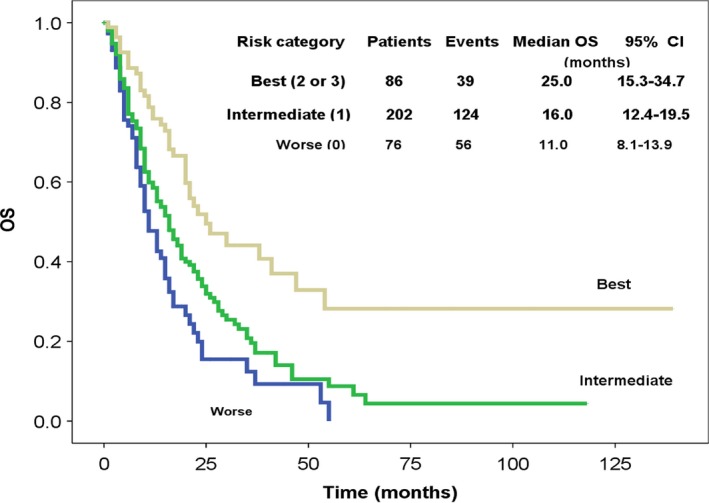
Kaplan–Meier curves of overall survival (OS) according to patients' TAF Score. After second‐line platinum‐based chemotherapy, patients with a TAF score of 2–3 had significant better survival than those scored 0 or 1 (*P* < 0.0001).

**Figure 3:**
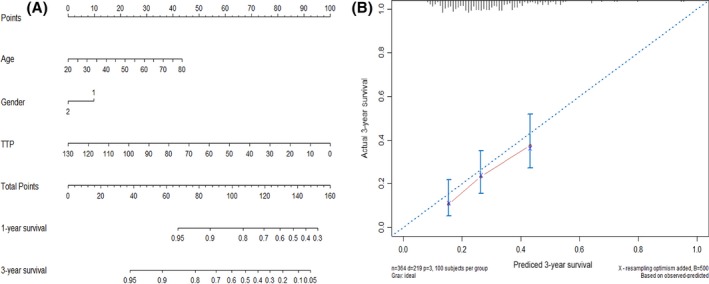
(A) A survival nomogram for advanced non‐small‐cell lung cancer patients who received platinum rechallenge as second‐line treatment. Age, patient's age when the second‐line chemotherapy stared; gender, 1 for male and 2 for female; time‐to‐progression (TTP), patient's TTP after first‐line platinum‐based chemotherapy. (B) The calibration curve for the prediction of 3‐year overall survival (OS). The nomogram predicted probability of OS is plotted on the *x* axis; the actual OS is plotted on the *y* axis.

Kaplan–Meier curves of OS according to TAF scores are shown in Figure 2. Median survival was 11.0, 16.0, and 25.0 months for TAF score of 0, 1, 2–3, respectively. Patients with more than 2 points of TAF score had the best OS after second‐line platinum‐based chemotherapy (median OS, 25.0 vs. 16.0 vs. 11.0 months, *P* < 0.0001). The estimated 2‐year survival rate was 15.5%, 33.8%, and 51.9% for the TAF scores of 0, 1, 2–3, respectively (*P* < 0.0001). A prognostic nomogram that integrated patient's age, gender, and TTP for OS is shown in Figure 3, with a C‐index of 0.623 (95% CI, 0.603–0.643).

## Discussion

Evidence of platinum rechallenge's clinical benefit as second‐line chemotherapy in advanced NSCLC is limited. Based on the findings reported by some randomized clinical trials, platinum did not add any significant benefit in terms of RR, PFS, or OS, as compared with pemetrexed or docetaxel alone in advanced NSCLC patients who had progressed after first‐line platinum‐doublet chemotherapy 13, 20. However, most of these studies did not access patients' TTP after first‐line chemotherapy, an interval that may distinguish platinum‐sensitive patients who benefit from second‐line platinum‐based chemotherapy. In fact, Smit et al. reported that in advanced NSCLC patients who had progressed after platinum‐based first‐line chemotherapy, those with a platinum‐free interval of more than 6 months had a significant better prognosis after second‐line treatment than the rest of the patient population 14. To date, no randomized clinical trials have been conducted in advanced NSCLC evaluating platinum rechallenge as second‐line treatment while classifying patients with their TTP after first‐line treatment.

This is the first study to establish a prognostic TAF score system for platinum sensitivity in NSCLC, labeling patients who may be suitable for platinum rechallenge as second‐line chemotherapy. A TAF score of 2 or 3 points means a good prognosis if these advanced NSCLC patients received platinum rechallenge after disease progression. In advanced NSCLC patients with ECOG PS 0–1, who previously received platinum doublet as first‐line chemotherapy and had disease progression, platinum‐based second‐line combination chemotherapy results in a median OS of 16.0 months, which is double that of single‐agent regimen reported by previous studies 3, 4, 6. We found that prognosis was better in patients younger than 60 years and in female patients, which is consistent with literature 2, 21. Patients in the >12‐month group have a significant better survival of 25.0 months, while patients in the 0‐ to 3‐month group face about twice the risk of dying. TTP after first‐line treatment, instead of the best effect of first‐line treatment or patients disease stage (IIIB vs. IV), significantly influences patients' long‐term survival. Platinum sensitivity can be ranked by the TAF score after first‐line platinum‐based chemotherapy. We found that compared with patients in the 0‐ to 3‐month group, patients in the >12‐month group received more cycles of chemotherapy. This is mainly because that most of patients with rapidly progressed disease did not show response to platinum‐based chemotherapy; some even progressed during the treatment. Meanwhile, patients who progressed more than 12 months usually show response well to platinum doublet, and most of them had completed four or more cycles of chemotherapy. Disease stage and tumor pathology were not well balanced in different groups: patients in the >12‐month group had more stage IIIB disease and more squamous cell carcinoma. However, prognosis of advanced NSCLC patients is not significantly conditioned by disease stage or tumor histology in our study.

It is generally considered that platinum will add treatment‐related toxicities when compared with single‐agent regimen, and that is why all our patients had ECOG PS of 0 or 1 before second‐line chemotherapy, instead of 0–2 as most of the previous studies had used for eligibility. Our results show that in these patients with good PS, platinum rechallenge as second‐line chemotherapy was well tolerated, and grade 3/4 toxicities were relatively infrequent. In particular, although patients in the >12‐month group received more cycles of platinum doublet as second‐line treatment, grade 3/4 neutropenia was only observed in 9.7% patients, and no grade 3/4 neuropathy occurred. Prolongation of OS after platinum rechallenge may not be obtained at the cost of deterioration of patient's quality of life.

Our study is limited by its retrospective nature, and the heterogeneity of patients' characteristics and treatment management. We did not analyze patients' genetic status of EGFR or anaplastic lymphoma kinase (ALK). Platinum doublet is the standard first‐line and second‐line treatment for mutation negative patients. Mutation‐positive patients who have received EGFR‐TKI or ALK inhibitor as first‐line treatment, also need platinum‐based chemotherapy after disease progression. Chemotherapy still plays an important role in the treatment of advance NSCLC. Our results are helpful for further evaluation of platinum rechallenge and single agent in advanced NSCLC patients according to their platinum sensitivity.

With these limitations in mind, a definite conclusion about the platinum‐based combined chemotherapy as second‐line treatment in NSCLC may not be stated. However, as the first and largest report of platinum sensitivity and platinum rechallenge in NSCLC, it provides relevant insight into the efficacy of platinum rechallenge in advanced NSCLC patients with different platinum sensitivity. In conclusion, our data suggest that for advanced NSCLC patients with good physical performance after first‐line platinum‐based chemotherapy, those who are female, younger than 60 years, and have progressed more than 12 months after previous treatment may benefit from platinum doublet as second‐line chemotherapy. Further investigation evaluating platinum rechallenge through randomized clinical trials should cautiously select these platinum‐sensitive patients.

## **Conflict of interest**

None declared.
